# The Nervous Origin of Diabetes

**Published:** 1901-02-16

**Authors:** 


### THE NERVOUS ORIGIN OF DIABETES.

Diabetes is one of those diseases which have often been said to be without a pathology, and there are many who, if asked what was its cause, would answer that it was due to a disturbance in the function of one organ or another.

Dr. Howship Dickenson,[1] however, in his teaching at St. George's Hospital, objects to the term "functional" as applied to such a disease. "To call a disease functional," he says, "is generally a confession of ignorance. In medicine we must all be materialists, and must seek structural change as at the bottom of every persistent perversion of action that is not accounted for by as persistent a perversion of external influences." He endeavours therefore to separate from among the various morbid conditions met with after death from diabetes, many of which are obviously the results of the disease, certain changes which he thinks it fair to regard as having had a casual influence in its production. Among these he specially points to certain changes in the brain (perivascular hæmorrhage and enlargement of certain groups of perivascular canals). We see, he says, in the brain a widely scattered disturbance of the relation between the arteries and their surroundings, not always in the same place nor to the same extent, but significant of morbid action. "Though we have not found out the initial source of the disturbance we cannot but suspect the vaso-motor centre as holding the key of the mystery." In the spinal cord also there are changes, chief among which may be mentioned dilatation of the central canal.

Of many of the morbid changes associated with diabetes the question arises whether they are effects or causes. It is not impossible that of those mentioned by Dr. Dickenson, as occurring in various parts of the body, many may be only effects, "but when we ascend to the brain we see . . . certain things which are difficult to explain except as the result of morbid processes primary to the brain itself. Such are the evidences of morbid action in the perivascular canals, more especially the frequent hæmorrhage by transudation." These "are not results of the saccharine state of the blood but are in some way connected with its cause." Turning now to the clinical relations of diabetes we see much to point to the probability of its having its origin in the nervous system. Grief, anxiety, terror may all be followed by diabetes so closely that there is no room for doubt as to their having occasioned it. It is well known as one result of commercial disaster, and it may be said truly that every panic on the Stock Exchange produces its crop of cases of diabetes. It has recently come to light that engine-drivers are especially subject to this disease, presumably from the anxious nature of their occupation, and Dr. Saundby states, on the authority of Mr. Herbert Page, surgeon to the London and North-Western Railway Company, that the engine-drivers on that railway become diabetic in nearly twice the proportion of the ordinary population. Among other causes the most prominent is heredity.

If we trace still further the clinical association of diabetes we see again how they relate to the nervous system. Thus we find loss of knee-jerk as an early symptom, one which occurs more frequently than is commonly supposed. Another point of contact between diabetes and the nervous system is to be found in the excess of the earthy phosphates, essentially phosphates of lime, in the urine in this disease. It is a matter of familiar observation that in certain states of the brain expressed by such terms as "worry" and "nervous irritation" the discharge of lime with the urine, virtually as phosphate is much increased. The same thing is found in diabetes, in severe and advanced examples of which disease the increase of urinary lime (practically all in the form of phosphate) may be termed extraordinary, since it is unexampled in any other malady. Still another link between diabetes and the nervous system is to be found in ts occasional association with insanity.

It must be confessed that we have not got to the bottom of the pathology of diabetes, but we must not cloak our ignorance by speaking of diabetes as a "functional disease." If a machine goes wrong there must be something wrong in the works or in the stuff supplied to it, or in the influences, motive-power and what not which are brought to bear upon it. If a mill were to discharge bran where it ought to discharge flour we should hardly be satisfied with the engineer who assured us that the mischief was functional; and if the perverted action which constitutes diabetes exists, notwithstanding that the material supplied to the organism and the circumstances by which it is surrounded are such as in other cases are attended with no such perversion, we must look into the organism for the error, for, though we may not succeed at once in finding it, it is surely there; and the associations of diabetes so ably discussed by Dr. Dickenson without doubt point strongly to the derangement, or rather the organic morbid changes on which diabetes depends, being situated in the nervous system.

[1] Lancet, Feb. 2.

